# Androgenic effects of aqueous and methanolic extracts of *Ficus asperifolia* in male Wistar rats

**DOI:** 10.1186/s12906-016-1547-5

**Published:** 2017-01-13

**Authors:** Pierre Watcho, Hermine Meli Watio, Modeste Wankeu-Nya, Esther Ngadjui, Patrick Deeh Defo, Pepin Alango Nkeng-Efouet, Telesphore Benoit Nguelefack, Albert Kamanyi

**Affiliations:** 1 Department of Animal Biology, Faculty of Science, Animal Physiology and Phytopharmacology Laboratory, University of Dschang, P.O. BOX 67, Dschang, Cameroon; 2 Department of Animal Organisms Biology, Laboratory of Animal Biology and Physiology, University of Douala, P.O. BOX 24157, Douala, Cameroon; 3 Department of Chemistry, Faculty of Science, University of Dschang, P.O. BOX 67, Dschang, Cameroon

**Keywords:** *Ficus asperifolia*, Androgen deficiency, Testosterone, Bicalutamide, Rat

## Abstract

**Background:**

Androgen deficiency is a clinical syndrome resulting from the inability of the testes to produce physiological levels of testosterone due to a disturbance occurring at one or more levels of the hypothalamic-pituitary-testicular axis. The present study was undertaken to evaluate the androgenic properties of aqueous and methanolic extracts of *Ficus asperifolia* on normal and castrated immature rats.

**Methods:**

Normal rats were treated either *per os* with aqueous or methanolic extract of *Ficus asperifolia* (100 mg/kg or 500 mg/kg b.w.), distilled water (10 ml/kg b.w.), 5% Tween 80 (10 ml/kg b.w.) or subcutaneously with testosterone propionate (0.5 mg/kg b.w.). Castrated rats were treated with plant extracts (100 mg/kg b.w. or 500 mg/kg b.w.) alone or with the co-administration of plant extracts and testosterone propionate (*s.c.,* 0.5 mg/kg b.w.) or bicalutamide (2 mg/kg b.w. per os). Animals were treated once a day during four weeks. Body weight growth and relative sexual organ weights were recorded at the end of each treatment. Some biomedical parameters were measured in the plasma (proteins, cholesterol), testes (cholesterol) and epididymis (proteins).

**Results:**

In normal rats, Ficus asperifolia significantly (*p* < 0.05) increased the relative weights of the testes and all sexual-dependent organs whereas total testicular cholesterol concentration was significantly (*p* < 0.05) decreased. In castrated groups, treatment with *Ficus asperifolia* was followed by an increase in the sexual organ weights, epididymal protein and prostatic acid phosphatase concentrations. The co-administration of testosterone and plant extracts significantly (*p* < 0.05) increased the weight of accessory sexual organs and epididymal protein contents. In the presence of bicalutamide (an anti-androgen), the sexual stimulating activity of *Ficus asperifolia* was diminished with remarkable effects on vas deferens weight (*p* < 0.05), plasma (*p* < 0.01) and epididymal (*p* < 0.05) protein contents.

**Conclusion:**

*Ficus asperifolia* possesses androgen-like activity through possible stimulation of cytoplasmic and/or nuclear receptors by the bioactive compounds found in its extracts.

## Background

Male reproduction is a complex process that involves the testes, epididymis, vas deferens, accessory sex glands, and associated hormones [1, 2]. Androgens are steroid hormones with an unequivocal role in sexuality. In general, androgens are essential for the development of the male external genitalia, the male secondary sexual characters and also in the regulation of erectile response [3]. Testosterone is the most important androgen secreted by the testis in humans [4]. A reduction in the level of testosterone at the early developmental phase results in the lack of virilization, sustained height increase without closure of the epiphysis, lack of pubertal growth spurt, incomplete sexual development and aspermia. In adulthood, it may result in the loss of libido and sexual activity [5]. The incidence of sexual dysfunction resulting from hormonal imbalance is estimated to be 20–25% with hypogonadism (primary and secondary) being the most frequent cause [3]. Testosterone replacement therapy has been found to be effective in restoration of these conditions [6]. These effects of testosterone in young and adult subjects can be prevented by several antiandrogens including finasteride, the 5-alpha reductase inhibitor [7] and bicalutamide, a non-specific antagonist androgen receptor [8]. The hormonal preparations currently used as a replacement therapy can produce adverse effects for instance on prostate gland, mammary gland, liver and cardiovascular functions [9]. A phytochemical with similar properties to that of the steroids that can bring about the changes necessary for restoration of general well-being, sexual interest and activity without producing the side effects will contribute significantly to the management of androgen deficiency. It has been reported that *Mondia whitei* [10, 11], *Bersama engleriana* [12], alkaloids found in *Alangium salviifolium* [13] or saponins isolated from *Tribulis terrestris* [14] increase weight of the sex accessory organs and the concentrations of testosterone and tissue proteins.

*Ficus asperifolia (F. asperifolia)* (L) Hook. ex Miq. belonging to the Moraceae family is a small or average size tree, terrestrial or epiphyte found in Senegal, Uganda, Tanzania, KwaZulu Natal (South Africa), Madagascar and Cameroon. Traditional medicine in the Western Region of Cameroon indicates that dried fruits of *Ficus asperifolia* are used to reverse some cases of infertility whereas the leaves are used as anthelmintic and purgative [15]. Previous results from our research group have shown that aqueous and methanolic fruit extracts of *F. asperifolia* enhance female fertility through an estrogenic pathway [16]. Although no work has been carried out to identify the effects of this medicinal plant on the male reproductive system, other extracts of the genus *Ficus*, which comprises a variety of about 900 species, shrubs, and vines commonly called figs, have been reported to increase sperm production and pH of sperm microenvironment in growing rat (*Ficus sycomorus*) [17], to favour spermatogenesis at low concentration or to treat azoospermia (*Ficus capensis*) [18, 19]. Based on these beneficial sexual reproductive activities of the genus *Ficus* and, since pro-sexual effects of many plant species are generally linked to the presence of some bioactive compounds, we hypothesized that, due to its contents in saponins and alkaloids [15, 20], *F. asperifolia* may also exhibit some sexual stimulant-like effects in males. The present study was therefore undertaken to evaluate the androgenic effects of aqueous and methanolic extracts of *F. asperifolia* in normal and androgen deficient immature rats. Since we also hypothesized that *F. asperifolia* could bind to the cytoplasmic or nuclear testosterone receptors, a powerful androgen receptor antagonist, bicalutamide, was also used in the present study.

## Methods

### Plant collection and preparation of extracts

Fresh fruits of *F. asperifolia* were collected in January 2014 at around 9 AM local time from trees in Batcham, Cameroon. Botanical identification was performed in the Cameroon National Herbarium (HNC) where a voucher N^o^ 338/15240/HNC has been deposited. The fruits were dried in the shade for 14 days and ground into powder. Two types of extracts were used in the study. In order to obtain an aqueous extract similar to the traditional preparation, 300 g of *F. asperifolia* were soaked in boiled distilled water (5.4 L). The mixture was allowed to cool at room temperature for 15 min and then filtered using Whatman paper N^o^ 3 and oven-dried to give 45.70 g of aqueous extract (yield of extraction, 15.23%; w/w based on the dried starting weight). To obtain the methanolic extract, 1 kg of *F. asperifolia* powder was soaked in 8 L of methanol (95%) for 24 h. The extract was filtered using Whatman paper N^o^ 3 and the filtrate was evaporated (78 °C) to dryness using a rotary evaporator; 25.64 g of dried methanolic extract were obtained giving an extraction yield of 2.56% (w/w based on the dried starting weight). For bioactivity investigations, the aqueous and methanolic extracts were dissolved in distilled water and 5% Tween 80 respectively.

### Animals

A total of 115 immature male Wistar rats (7-8 weeks, 100-150 g body weight) were obtained from the animal house of the Animal Biology Department of Dschang University, Cameroon. They were housed in groups (four rats per cage), under natural LD cycle and with free access to food and water. The experiments were performed in accordance with the internationally accepted standard ethical guidelines for laboratory animal use and care as described in the European Community guidelines; EEC Directive 86/609/EEC, of the 24th November 1986 [21].

Castration was performed following the techniques previously described [22]. The animals were anaesthetized by intraperitoneal injection of Diazepam (10 mg/kg b.w.) followed 10 min later by Ketamine (50 mg/kg b.w.). After the onset of anaesthesia, the scrotal area was clipped and prepared aseptically. A 1/2 cm incision was made in the scrotal sac. Each testis was delivered separately through the scrotal incision. Once exteriorized, the testis was removed by severing the vas deferens and spermatic artery. The incision was sutured in an interrupted pattern and finally, an intramuscular injection of penicillin G (2000 IU/kg b.w./day/3 days). One week later, the castrated animals were used for the experiments.

### Drugs

Testosterone propionate (Schering AG, Germany), Bicalutamide (Astra Zeneca, Belgium), Diazepam (Renaudin, France), Ketamine (Rotex Medica, Germany) and Penicillin G (Clarion Medicals, Nigeria) were of analytical grade.

### Animals grouping and treatment

#### Effects of *F. asperifolia* and testosterone in normal immature male rats

Thirty five (35) normal (non castrated) male rats were randomly divided into 7 groups of 5 rats each and treated with one of the following: distilled water (10 ml/kg b.w.); 5% Tween 80 (10 ml/kg b.w.); testosterone (0.5 mg/kg b.w.); aqueous extract of *F. asperifolia* (100 mg/kg b.w. or 500 mg/kg b.w.); methanolic extract of *F. asperifolia* (100 mg/kg b.w. or 500 mg/kg b.w.).

### Effects of drugs in castrated male rats

#### Effects of aqueous and methanolic extracts of *F. asperifolia*

Thirty immature castrated male rats were randomly divided into 6 groups of 5 animals each and treated with one of the following: distilled water (10 ml/kg b.w.); testosterone (0.5 mg/kg b.w.); aqueous extract of *F. asperifolia* (100 mg/kg b.w. or 500 mg/kg b.w.); methanolic extract of *F. asperifolia* (100 mg/kg b.w. or 500 mg/kg b.w.).

#### Effects of co-administration of *F. asperifolia* and testosterone

In order to ascertain whether *F. asperifolia* could potentiate the androgenic effect of exogenous testosterone, twenty five immature castrated male rats were partitioned into 5 groups of 5 animals each and treated with one of the following: 5% Tween 80 (10 ml/kg b.w.) plus testosterone (0.5 mg/kg b.w.); testosterone plus aqueous extract of *F. asperifolia* (100 mg/kg b.w. or 500 mg/kg b.w.); testosterone plus methanolic extract of *F. asperifolia* (100 mg/kg b.w. or 500 mg/kg b.w.).

#### Effects of co-administration of *F. asperifolia* and bicalutamide

To verify the use of cytoplasmic or nuclear androgen receptors by *F. asperifolia,* twenty five castrated rats were divided into 5 groups of 5 animals each and treated with one of the following: bicalutamide (2 mg/kg b.w.) plus testosterone (0.5 mg/kg b.w.); bicalutamide (2 mg/kg b.w.) plus aqueous extract of *F. asperifolia* (100 mg/kg b.w. or 500 mg/kg b.w.); bicalutamide (2 mg/kg b.w.) plus methanolic extract of *F. asperifolia* (100 mg/kg b.w. or 500 mg/kg b.w.).

All test substances were administered once a day for 4 weeks. Testosterone was given subcutaneously while *F. asperifolia*, bicalutamide and the solvents were given orally at the volume 10 ml/kg b.w.

### Sacrifice and biochemical measurements

One day after the last treatment (day 29), animals were weighed and sacrificed under diazepam/ketamine anesthesia; abdominal artery blood was collected into heparinized tubes and centrifuged to obtain the plasma. Testes (if present), vas deferens, epididymis, ventral prostate and seminal vesicles were then removed, cleared of fat and weighed. Total proteins and cholesterol were determined in the plasma. Tissues from each rat were kept at -20 °C until assayed for total epididymal protein [23], prostatic acid phosphatase [24] and total testicular cholesterol (commercial kit, INMESCO, GmbH, Germany).

### Statistical analysis

Data are reported as the mean plus standard error of mean (SEM). One-way analysis of variance (ANOVA I) followed by post-hoc LSD were used to analyze statistical difference among groups. Comparisons with *p* values < 0.05 were considered to be statistically significant. The statistical tests were performed with STATISTICA Version 8.

## Results

### Effects of treatments on immature normal rats

A daily subcutaneous injection of testosterone propionate (0.5 mg/kg b.w.) for 4 consecutive weeks was followed by a significant (*p* < 0.01) increase in body weight and relative weights of the accessory organs with an expressive effect on the seminal vesicles (*p* < 0.001) and a remarkable accumulation of testicular cholesterol (*p* < 0.001).

After 4 weeks of oral administration of the plant extracts (aqueous and methanolic), there was an increase in the body weight in all *F. asperifolia-*treated groups compared to control animals. Relative weights of testes, epididymis, vas deferens, ventral prostate and seminal vesicles were significantly (*p* < 0.05) increased. The methanolic extract (500 mg/kg b.w.) appeared to be more effective than the aqueous extract (Table 1). Total protein and cholesterol contents of plasma were statistically unchanged whereas the epididymal protein level was increased (*p* > 0.05). *F. asperifolia* treatment resulted in significant (*p* < 0.05) decrease in testicular cholesterol and prostatic acid phosphatase (Table 2).

**Table 1:** Effects of testosterone and, aqueous and methanolic extracts of *F. asperifolia* on body weight variation and relative organ weights of immature normal rats after four weeks of treatment

Groups	Body weight (g)		Body weight variation (%)	Relative organ weights (mg/100 g)				
	Initial	Final		Testes	Epididymis	Vas deferens	Ventral prostate	Seminal vesicles
Distilled water (10 ml/kg b.w.)	120.00 ± 6.4	173.40 ± 8.84	44.43 ± 1.94	1087.85 ± 20.38	232.78 ± 21.60	63.32 ± 5.05	41.37 ± 7.23	186.47 ± 30.51
5% Tween 80 (10 ml/kg b.w.)	118.80 ± 4.35	168.00 ± 6.77	41.51 ± 3.33	1099.65 ± 71.13	231.52 ± 27.78	57.65 ± 5.44	45.63 ± 7.42	145.48 ± 26.45
Testosterone (0.5 mg/kg b.w.)	120.40 ± 5.87	210.20 ± 12.3	74.41 ± 4.59**	416.25 ± 53.92***	197.87 ± 7.31	66.32 ± 1.68	66.59 ± 11.06	484.98 ± 27.42***
*F. asperifolia*								
Aqueous extract								
100 mg/kg b.w.	121.20 ± 4.03	176.80 ± 7.11	45.81 ± 2.62^≠≠^	1098.54 ± 77.69^≠≠≠^	338.34 ± 29.42**^≠≠≠^	81.64 ± 6.00*^≠^	76.72 ± 16.10*	273.40 ± 38.99*^≠≠≠^
500 mg/kg b.w.	124.80 ± 7.08	187.80 ± 9.49	51.05 ± 4.91^≠^	1161.92 ± 75.33^≠≠≠^	310.56 ± 23.81*^≠≠^	64.34 ± 2.66	64.63 ± 5.09	243.13 ± 19.96^≠≠≠^
Methanolic extract								
100 mg/kg b.w.	120.00 ± 6.38	190.60 ± 9.66	60.16 ± 10.40	1189.18 ± 53.47^≠≠≠^	327.13 ± 24.35*^≠≠^	70.77 ± 4.27	96.42 ± 11.72**	307.49 ± 28.97**^≠≠≠^
500 mg/kg b.w.	123.40 ± 7.81	194.20 ± 6.45	59.95 ± 11.47	1279.15 ± 14.60*^≠≠≠^	340.21 ± 32.27**^≠≠≠^	72.76 ± 5.80	99.81 ± 13.00***^≠α^	308.94 ± 29.35**^≠≠≠^

All values: Mean ± SEM; Number of rats per group = 5; *p < 0.05; ** *p* < 0.01; ***p < 0.001 compared to distilled water; ^≠^ *p* < 0.05; ^≠≠^ *p* < 0.01; ^≠≠≠^ *p* < 0.001 compared to testosterone; ^α^ *p* < 0.05 significantly different at equal dose of plant extracts

**Table 2:** Effects of testosterone and, aqueous and methanolic extracts of *F. asperifolia* on total proteins, total cholesterol and prostatic acid phosphatase in normal immature rats after four weeks of treatment

Groups	Total proteins		Total cholesterol		Prostatic acid phosphatase (U/g)
	Epididymis (mg/ml)	Plasma (mg/ml)	Testes (mg/g)	Plasma (mg/dl)	
Control (Distilled water, 10 ml/kg b.w.)	85.70 ± 8.82	61.18 ± 3.33	0.86 ± 0.21	7.42 ± 0.23	2.55 ± 0.63
5% Tween 80 (10 ml/kg b.w.)	83.44 ± 15.20	54.58 ± 2.28	1.77 ± 0.30	6.30 ± 1.14	2.37 ± 0.59
Testosterone (0.5 mg/kg b.w.)	72.88 ± 14.20	48.50 ± 6.43*	1.03 ± 0.19**	7.31 ± 1.1	1.00 ± 0.13**
*F. asperifolia*					
Aqueous extract					
100 mg/kg b.w.	121.82 ± 5.77^≠^	60.89 ± 1.55^≠^	0.59 ± 0.19	7.01 ± 1.07	0.93 ± 0.18**
500 mg/kg b.w.	101.05 ± 22.51	53.06 ± 3.35	0.39 ± 0.09^≠^	8.05 ± 0.45	1.19 ± 0.16*
Methanolic extract					
100 mg/kg b.w.	107.37 ± 17.58	60.61 ± 1.65^≠^	0.16 ± 0.07**^≠≠^	7.78 ± 0.68	0.74 ± 0.11**
500 mg/kg b.w.	109.45 ± 21.03	63.04 ± 1.98^≠≠α^	0.34 ± 0.07*^≠≠^	7.26 ± 0.36	1.39 ± 0.25*

All values: Mean ± SEM; Number of rats per group =5; *p < 0.05; **: *p* < 0.01 compared to distilled water; ^≠^: *p* < 0.05; ^≠≠^: *p* < 0.01 compared to testosterone group; ^α^: *p* < 0.05 at equal dose of plant extracts

### Effects of drugs in immature castrated rats

#### Effects of *F. asperifolia* extracts in immature castrated rats

Castration was generally followed by a significant drop in the weights of the epididymis, vas deferens, ventral prostate and seminal vesicles when compared to non-castrated controls (normal rats) (Table 3). In androgen-deficient animals, a daily subcutaneous injection of testosterone propionate (0.5 mg/kg b.w.) for 4 consecutive weeks always brought out, as expected, important changes as evidenced by the body mass increase (65.08 ± 4.69%) and the significantly (*p* < 0.001) growth of all androgen-dependent organs. These sexual stimulating effects of testosterone propionate were more expressed on seminal vesicles and ventral prostate where an increase of 2343.3% and 1417.40% was recorded respectively.

**Table 3:** Effects of various treatments on body weight variation and relative organ weights of immature castrated rats after four weeks of treatment

Treatments	Body weight (g)		Body weight variation (%)	Relative organ weights (mg/100 g)			
	Initial	Final		Epididymis	Vas deferens	Ventral prostate	Seminal vesicles
Normal plus distilled water (10 ml/kg b.w.)	120.00 ± 6.4	173.40 ± 8.84	44.63 ± 1.94	232.78 ± 21.60	63.32 ± 5.05	41.37 ± 7.23	186.47 ± 30.51
Castrated plus distilled water (10 ml/kg b.w.)	124.8 ± 7.53	179 ± 14.89	43.17 ± 7.56	42.47 ± 5.94^C^	16.06 ± 2.15^C^	5.69 ± 0.54^C^	10.00 ± 1.81^C^
Castrated plus testosterone (0.5 mg/kg b.w.)	125.6 ± 3.41	207 ± 5.5	65.08 ± 4.69***	143.10 ± 2.67***	50.45 ± 3.52***	86.34 ± 2.93***	244.53 ± 23.11***
5% Tween 80 (10 ml/kg b.w.) + testosterone	120 ± 5.91	179.8 ± 15.16	49.15 ± 6.05**	141.15 ± 13.29***	51.36 ± 3.90***	68.83 ± 14.66***	243.75 ± 58.42***
Bicalutamide (2 mg/kg b.w.) + testosterone	127.4 ± 5.63	196.8 ± 15.39	54.30 ± 7.98	83.93 ± 7.98	38.30 ± 3.79^##^	26.76 ± 0.82^###^	112.54 ± 18.45^###^
*F. asperifolia*							
Aqueous extract							
100 mg/kg b.w.	120 ± 6.44	167 ± 5.89	39.73 ± 2.82	77.20 ± 5.00*	18.89 ± 0.91	10.35 ± 1.90	19.11 ± 2.24
500 mg/kg b.w.	121 ± 8.28	161.8 ± 9.29	34.12 ± 1.90	61.76 ± 10.99	25.09 ± 3.72	11.06 ± 1.75	19.60 ± 3.77
Methanolic extract							
100 mg/kg b.w.	121.6 ± 5.9	174.6 ± 7.69	43.76 ± 2.20	38.62 ± 1.04	12.67 ± 0.39	6.74 ± 0.72	17.18 ± 1.87
500 mg/kg b.w.	120.8 ± 1.61	169.8 ± 9.04	40.50 ± 0.75	40.18 ± 6.76	15.89 ± 2.18	8.074 ± 1.50	21.28 ± 4.49
Testosterone (0.5 mg/kg b.w.) plus aqueous extract							
100 mg/kg b.w.	125.8 ± 5.06	184.6 ± 9.55	46.50 ± 2.93^###●^	162.37 ± 8.60^●●●^	54.85 ± 1.91^●●●^	100.00 ± 8.43^●●●^	322.15 ± 30.10^#●●●^
500 mg/kg b.w.	126.2 ± 3.93	187.4 ± 5.03	48.62 ± 1.91^###^	165.86 ± 9.65^●●●^	61.51 ± 4.03^#●●●^	101.38 ± 2.66^●●●^	325.08 ± 16.12^#●●●^
Testosterone plus methanolic extract							
100 mg/kg b.w.	120.4 ± 5.91	175.6 ± 8.32	45.96 ± 2.39^###^	182.87 ± 16.88^##●●●^	72.86 ± 5.29^###●●●^	162.15 ± 17.84^###●●●^	544.91 ± 62.23^###●●●^
500 mg/kg b.w.	122 ± 4.51	185 ± 4.16	52.12 ± 3.95^≠^	137.50 ± 8.16^●●●^	65.16 ± 3.99^##●●●^	146.33 ± 11.15^###●●●^	455.53 ± 36.05^###●●●^
Bicalutamide (2 mg/kg b.w.) plus aqueous extract							
100 mg/kg b.w.	125.8 ± 5.79	183.8 ± 5.49	47.64 ± 5.67	79.35 ± 7.16	23.83 ± 2.64	6.315 ± 1.23	31.90 ± 4.28
500 mg/kg b.w.	122.2 ± 5.3	183.4 ± 9.36	50.07 ± 1.99	34.74 ± 7.39	10.29 ± 2.64^●^	1.31 ± 0.36	13.21 ± 1.85
Bicalutamide plus methanolic extract							
100 mg/kg b.w.	120.8 ± 4.89	161.6 ± 6.27	34.12 ± 4.71	33.34 ± 3.46	15.57 ± 1.90	4.79 ± 0.47	15.68 ± 2.34
500 mg/kg b.w.	120 ± 5.97	167 ± 7.83	39.40 ± 3.63	43.35 ± 3.41	19.01 ± 0.67	7.63 ± 0.75	17.45 ± 1.93

All values: Mean ± SEM; Number of rats per group =5; ^C^: *p* < 0.001 compared to normal rats; *: *p* < 0.05; ***: *p* < 0.001 compared to castrated distilled water treated rats; ^#^: *p* < 0.05; ^##^: *p* < 0.01; ^###^: *p* < 0.001 compared to testosterone group; ^●^: *p* < 0.05; ^●●^: *p* < 0.01; ^●●●^: *p* < 0.001 compared to rats treated only with plant extracts

With regard to castrated controls, rats exposed to the plant extracts showed no increase in the body weight after 4 weeks of treatment. However, a trend to an increase in the relative organ weights was observed in all *F. asperifolia-*treated groups compared to the respective control. The dose of 100 mg/kg of aqueous extract increased significantly (*p* < 0.05) the weight of the epididymis. Similar to testosterone propionate, *F. asperifolia* increased the protein contents of epididymis and the prostatic acid phosphatase compared to castrated rats receiving distilled water. Level of plasma proteins was also increased in animals treated with the methanolic extract of *F. asperifolia* (*p* < 0.05) (Table 4).

**Table 4:** Effects of various treatments on total proteins and prostatic acid phosphatase in castrated rats after four weeks of treatment

Treatments	Total proteins		Prostatic acid phosphatase (U/g)
	Epididymis (mg/g)	Plasma (mg/ml)	
Normal plus distilled water (10 ml/kg b.w.)	85.70 ± 8.82	61.18 ± 3.33	2.55 ± 0.63
Castrated plus distilled water (10 ml/kg b.w.)	52.03 ± 11.18	58.99 ± 3.06	0.21 ± 0.04^C^
Castrated plus testosterone (0.5 mg/kg b.w.)	75.68 ± 16.84	64.92 ± 1.88	0.78 ± 0.10*
5% Tween 80 (10 ml/kg b.w. plus testosterone	62.13 ± 8.90	62.15 ± 5.39	0.75 ± 0.24
Bicalutamide (2 mg/kg b.w.) plus testosterone	45.33 ± 9.22	56.10 ± 4.24	0.37 ± 0.17
Castrated plus aqueous extract			
100 mg/kg b.w.	91.84 ± 14.57	64.62 ± 3.01	0.59 ± 0.20
500 mg/kg b.w.	77.30 ± 24.02	66.26 ± 3.10	0.28 ± 0.06
Castrated plus methanolic extract			
100 mg/kg b.w.	59.24 ± 15.86	78.18 ± 2.80***	0.26 ± 0.08
500 mg/kg b.w.	67.82 ± 20.63	70.27 ± 4.93*	0.54 ± 0.11
Testosterone (0.5 mg/kg b.w.) plus aqueous extract			
100 mg/kg b.w.	108.73 ± 22.76	45.27 ± 2.51^###●●●^	0.51 ± 0.04
500 mg/kg b.w.	127.87 ± 22.14^#●^	50.93 ± 1.93^##●●^	0.58 ± 0.09
Testosterone (0.5 mg/kg b.w.) plus methanolic extract			
100 mg/kg b.w.	162.83 ± 33.53^###●●●^	62.30 ± 4.87^●●^	0.35 ± 0.10
500 mg/kg b.w.	105.20 ± 16.93	57.15 ± 1.14^●●^	0.35 ± 0.09
Bicalutamide (2 mg/kg b.w.) plus aqueous extract			
100 mg/kg b.w.	44.34 ± 10.49	70.29 ± 3.74	0.11 ± 0.05
500 mg/kg b.w.	22.40 ± 7.55^●^	64.24 ± 3.86	0.24 ± 0.06
Bicalutamide (2 mg/kg b.w.) plus methanolic extract			
100 mg/kg b.w.	29.62 ± 7.43	59.26 ± 2.20^●●●^	0.15 ± 0.06
500 mg/kg b.w.	26.55 ± 10.15	53.73 ± 2.50^●●^	0.18 ± 0.03

All values: Mean ± SEM; Number of rats per group =5; ^C^: *p* < 0.001 compared to normal rats; *: *p* < 0.05; ***: *p* < 0.001 compared to distilled water treated rats; ^#^: *p* < 0.05; ^##^: *p* < 0.01; ^###^: *p* < 0.001 compared to testosterone group; ^●^: *p* < 0.05; ^●●^: *p* < 0.01; ^●●●^: *p* < 0.001 compared to rats treated only with plant extracts

### Effects of co-administration of *F. asperifolia* extracts with testosterone

After the sequential treatment of rats with plant extracts and the exogenous androgen, testosterone propionate, the body weight was significantly (*p* < 0.05) reduced whilst the weights of the epididymis, vas deferens, ventral prostate and seminal vesicles were increased in all groups when compared to rats treated only with testosterone propionate (Table 3). In comparison to animals treated only with plant extracts, this treatment significantly (*p* < 0.001) increased the weights of all organs at all doses although the increase in body weight was significant only with the aqueous extract-treated group. At equal dose, the methanolic extract was more efficient than the aqueous extract. The level of epididymal protein also increased significantly (*p* < 0.05). The plasma protein content was significantly (*p* < 0.01) reduced while the observed reduction in prostatic acid phosphatase concentration was not significant when compared either to animals treated with testosterone or plant extracts (Table 4).

### Effects of co-administration of *F. asperifolia* extracts with bicalutamide

The evidence that *F. asperifolia* promotes growth of accessory sex organs and potentiates the androgenic effects of testosterone in androgen-deficient animals was clearly shown in this work. The co-administration of bicalutamide, an androgen receptor antagonist, along with aqueous or methanolic extract of *F. asperifolia*, resulted in a decrease in the weights of the sex accessory organs with a significant (*p* < 0.05) effect recorded at the dose of 500 mg/kg b.w. of aqueous extract on vas deferens. Epididymal and plasma protein were significantly (*p* < 0.05) reduced (Table 4). The reduction in prostatic acid phosphatase concentration was not significant while the body weight remained unchanged when compared to rats receiving only plant extracts (Table 4).

## Discussion

The evaluation of parameters such as sexual organs/body weight ratio, concentrations of protein, cholesterol as well as acid phosphatase activity can give useful information on the androgenic and/or anti-androgenic potential of a substance [25].

In the present study, oral administration of aqueous and methanolic extracts of *F. asperifolia* at the doses of 100 and 500 mg/kg b.w. resulted in a body weight gain in gonado-intact treated animals. The weights of the reproductive organs also increased significantly. Steroidgenesis is one of the causes of increased body and sexual organ weight. An increase in these parameters could be regarded as a biological indicator for effectiveness of the plant extract in improving the synthesis of steroidal hormones [26]. Androgenic steroids are essential for male development and the major androgen is testosterone secreted from testes. In rats, any increase in serum testosterone or treatment with androgens or androgen-like substances is associated with increased secretory activity of sexual organs [27]. As such, the observed increase in tissue total proteins in the present study suggests an important anabolic effect of the plant [25] which is further supported by the decreases in the intratesticular cholesterol and prostatic acid phosphatase levels. Cholesterol is considered as the major substrate for steroidogenesis and its testicular drop may reflect a conversion into testosterone under the control of luteinizing hormone [28, 29]. These results are similar to those obtained by [30, 31] following the oral administration of extracts from *Nymphaea lotus* flowers and *Cocculus hirsutus* leaves respectively to male rats. After puberty, the plasma level of testosterone is generally about 0.6 μg/dl [3] and this amount controls the growth and secretions of the accessory sex organs. Therefore, the observed decreased in the prostatic acid phosphatase level could possibly be due to the increase in blood testosterone concentration. Alkaloids and saponins revealed in the extracts of *F. asperifolia* [15] may account for its androgenic potentials. Many studies have demonstrated that alkaloids elevate testicular cholesterol in male testes while saponins stimulate endogenous testosterone levels probably by raising the level of luteinizing hormone or binding to enzymes involved in steroidogenesis [32, 33].

In order to confirm this androgenic effect of *F. asperifolia*, immature androgen-deficient animal models were used. It is generally believed that castration is strictly associated with a reduction in the weights and secretion of sexual-dependant organs such as prostate and seminal vesicles [34]. As expected, a subcutaneous injection of testosterone propionate almost alleviated these deficiencies. Similarly after four weeks of treatment, *F. asperifolia* increased the organs to body weight ratio, level of protein (in plasma and epididymis) and prostatic acid phosphatase. These results clearly suggest that *F. asperifolia* possesses an androgen-like activity with can further be supported by the drop observed in the relative weights of the sexual-dependent organs in the untreated castrated animals [22]. Similar androgenic effects were reported with *Tribulis terrestris* [35]*.* Co-administration of testosterone with *F. asperifolia* was followed with an over-expression of the effects of testosterone therefore suggesting a potentiating effect of the plant. Similar effects were observed after a combined administration of testosterone propionate and low dose (100 mg/kg b.w.) of *Leptadenia hastata* aqueous extract on immature castrated rats [36].

Substances with androgenic potentials can be classified into three categories: those that stimulate the biosynthesis of testosterone through central or testicular pathway; those that promote the conversion of testosterone into dihydrotestosterone (DHT) and those that stimulate the androgen receptor [37]. In order to ascertain the androgenic pathway of *F. asperifolia*, the effects of aqueous and methanolic extracts were examined in the presence of bicalutamide, a potent specific non-steroidal androgen receptor antagonist which competitively inhibits the binding of androgens to the androgen receptor [38]. Reduction of the effects of *F. asperifolia* in the presence of bicalutamide denotes the binding of *F. asperifolia* bioactive compounds to the cytoplasmic and/ or nuclear androgen receptors. However, results of the test also revealed that bicalutamide, in a similar manner to its effects on testosterone, did not completely abolish the action of *F. asperifolia*. It could then be proposed that *F. asperifolia* may also require other androgen pathways to completely exhibit its androgen-like activity. Therefore, the further investigation of the actions of *F. asperifolia* in animals co-treated either with a gonadotropic antagonist or an inhibitor of 5-alpha reductase is highly needed.

## Conclusion

Present results show that *F.asperifolia* possesses androgen-like effects through the partly stimulation of cytoplasmic and/or nuclear androgenic receptor by the bioactive compounds found in its extracts. However, further studies are required for a better understanding of the mechanism of *F. asperifolia* and also for its further use against some male sexual troubles including erectile dysfunction.

## Abbreviation

ANOVA: Analysis of variance
DHT: Dihydrotestosterone
EEC: European Community guidelines
F. asperifolia: *Ficus asperifolia*
HNC: Cameroon National Herbarium
LSD: Low statistical difference
